# Tracking changes in touch desire and touch avoidance before and after the COVID-19 outbreak

**DOI:** 10.3389/fpsyg.2022.1016909

**Published:** 2022-12-01

**Authors:** Yusuke Ujitoko, Takumi Yokosaka, Yuki Ban, Hsin-Ni Ho

**Affiliations:** ^1^NTT Communication Science Laboratories, Nippon Telegraph and Telephone Corporation, Atsugi, Japan; ^2^Graduate School of Frontier Sciences, The University of Tokyo, Chiba, Japan

**Keywords:** touch desire, touch avoidance, COVID-19, Twitter, skin hunger

## Abstract

Touch is essential for survival, social bonding, and overall health. However, the COVID-19 pandemic calls for an abrupt withdrawal from physical contact, and the prolonged lockdown has left many people in solitude without touch for months. This unprecedented dissociation from touch has cast a shadow on people's mental and physical well-being. Here we approached the issue by examining COVID-19's impact on people's touch attitudes. We analyzed people's desire and avoidance for animate and inanimate targets based on large-scale Japanese Twitter posts over an 8-year span. We analyzed the impact of the COVID-19 outbreak with the difference-in-differences estimation method, which can estimate the impact while accounting for other changes over time such as seasonality or long-term effects. As a result, we found that people's desire for touching the human body and pet animals increased significantly after the COVID-19 outbreak and remained high afterward. In contrast, the avoidance of touching everyday objects (e.g., doorknobs and money) increased immediately after the outbreak but gradually returned to the pre-COVID-19 levels. Our findings manifest the impact of COVID-19 on human touch behavior. Most importantly, they highlight the sign of “skin hunger,” a public health crisis due to social distancing, and call attention to the trend that people are becoming less aware of infection control as COVID-19 persists.

## 1. Introduction

Since the global outbreak of the COVID-19 pandemic, large-scale interventions, such as stay-at-home requests and social distancing rules, have affected every aspect of life. People's attitudes toward touch, such as touch desire and touch avoidance have shown dramatic signs of change. Studies have shown that during the COVID-19 pandemic, the frequency of interpersonal touch, i.e., touch that conveys emotion and is used for social communication, has decreased significantly (Rognon et al., 2021; von Mohr et al., 2021) and that people have started to express a greater desire for having physical contact with their loved ones (von Mohr et al., 2021). These changes in desires toward touch have a considerable impact on people's well-being. People with limited physical contact with their closest acquaintances, such as family members and partners, tend to have more anxiety and loneliness, poorer mental health, and a lower loneliness tolerance (von Mohr et al., 2021). Furthermore, a life of touch deprivation might put some people in a condition of “skin hunger,” a public health crisis that has been highlighted in major mass media (Maham Hasan, 2021). “Skin hunger” may impact all aspects of our health and has been associated with increases in stress, anxiety and depression (Durkin et al., 2021).

The pandemic is also expected to influence people's touch attitudes toward handling objects and explorating the environment with the hands. While the majority of transmissions of COVID-19 occur as a result of infected people emitting droplets when they cough or breathe, contact with contaminated surfaces has also been suggested as a possible means of transmission (Van Doremalen et al., 2020). Previous studies have shown that people are reluctant to touch objects that may be a source of infection (Oum et al., 2011; Iwasa et al., 2020). Accordingly, it is expected that people would have a strong sense of avoidance toward surfaces or objects such as doorknobs and handrails because other people might have touched them. Although touch avoidance would prevent infection, at the same time, people might sacrifice the benefits of manual exploration. People rely on not only visual information but also touch information to plan and act in the external world. Touch has been shown to facilitate material recognition (Tiest, 2010; Ho, 2018), memory retention in learning (Novak et al., 2020; Novak and Schwan, 2021), and decision-making in consumer behavior (Peck and Childers, 2003). Therefore, it is possible that the benefits of manual exploration are becoming harder to obtain in the current COVID-19 pandemic.

In the present study, we conducted a Twitter survey to track the changes in people's touch desire and touch avoidance before and after the COVID-19 pandemic in Japan. Understanding how people's touch desire and avoidance change as a result of the COVID-19 pandemic continues is essential not only for the scientific understanding of human behavior but also for assisting policy-making for the control of the epidemic. Moreover, recognizing people's touch desire and touch avoidance, both the degree and the target, will contribute to public health monitoring and the development of ICT technology. For example, tracking touch desire will help in monitoring the public health crisis of “skin hunger” during the pandemic (Field et al., 2020) and contribute to the development of a technology that allows people to engage in social touch at a distance (Huisman, 2017).

## 2. Methods

We chose Twitter for our analysis to aggregate the self-report of what people desire to touch and avoid touching. Twitter has accumulated a large amount of users' spontaneous reports (called “tweets”) about their own intention or behavior at various timings throughout the days. The public API provided by Twitter allowed us to obtain a considerable quantity of user-generated tweets in the form of a large-scale text corpus. In contrast to a questionnaire-based investigation (Rognon et al., 2021; von Mohr et al., 2021), the posts on Twitter are not prompted by investigators so there are few investigators-led effects on the posts. The usability of Twitter analytics has been demonstrated by a number of studies that have examined people's thinking and behavior (Bollen et al., 2011; Golder and Macy, 2011; Tumasjan et al., 2011; Ujitoko et al., 2021) and how people's interest in the COVID-19 pandemic has shifted (Wicke and Bolognesi, 2021). Note that there are several limitations on the use of Twitter such as the use of specific social media or skewed populations of Twitter users in Japan. Further, there could be noisy text on Twitter since text can be posted by anyone or even by automated posting software. Thus, in this paper, we aim to get insights into the effect of COVID-19 on touch attitudes by collecting and analyzing data collected via Twitter while carefully handling noise within the data.

**Figure 1:**
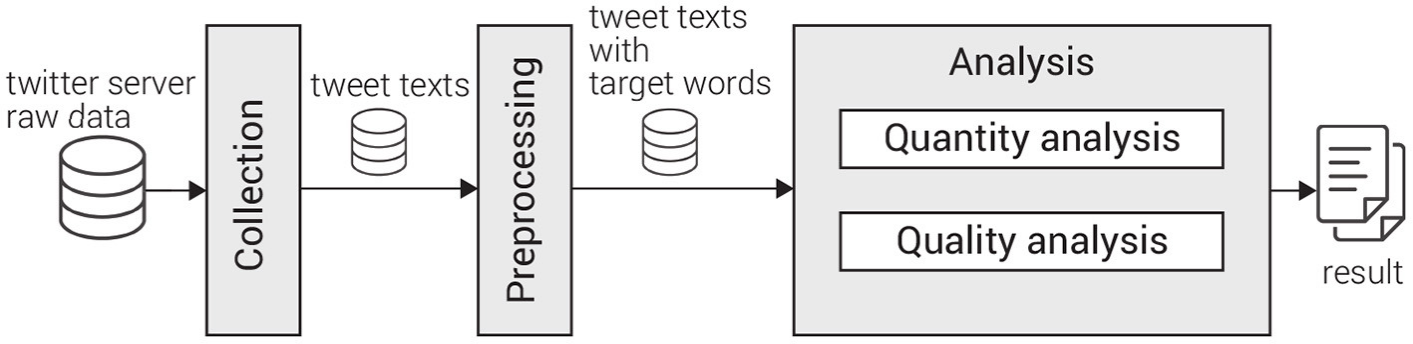
The flow of investigation comprises collection, preprocessing, and analysis stages.

The flow of our investigation consisted of collection (Section 2.1), preprocessing (Section 2.2), and analysis stages as shown in Figure 1. As analysis, we conducted quantity analysis (Section 2.3) and quality one (Section 2.4).

### 2.1. Data collection

Japanese tweet texts posted from February 1st, 2013 to January 31st, 2021, were collected. We collected tweet text expressing touch desire or touch avoidance from Twitter. We used a query “want to touch (‘sawaritai' in Japanese)” to collect tweet texts representing touch desire. One example of touch desire is “I want to touch a cat,” in which a cat is the target of touch desire. We also used the query “don't want to touch (‘sawaritakunai')” to collect tweet texts representing touch avoidance. One example of touch avoidance is “I don't want to touch the doorknob,” in which the doorknob is the target of touch avoidance. In summary, we fed two Japanese phrases “sawaritai” (“want to touch”) and “sawaritakunai” (“don't want to touch”). Though we also tried other query candidates by using other verbs (e.g., “like to touch” or “feel good to touch”), it turned out that those queries only resulted in a small number of tweets, which were less than 2% when using the query “want to touch” at certain 30 days (Ujitoko et al., 2021). Therefore, we decided the query “want to touch” and “don't want to touch,” which extracted a large number of tweets, would be the best for time-series analysis. See the third column in Table 1 for the total number of collected tweets. The difference in the number between the touch desire and touch avoidance might suggest the difference in interest for each touch attitude in daily life.

**Table 1:** Number of tweets and extracted target words.

**Query in Japanese**	**Meaning in English**	**Number of** **collected tweets**	**Number of** **with target words**	**Number of** **with target words** **after noisy target** **words exclusion**
Sawaritai	Want to touch	1,249,695	312,008	215,446
Sawaritakunai	Don't want to touch	193,566	27,703	12,848

Then, a series of exclusion processes was performed. To avoid the double counting of tweets posted by a bot (a bot is a type of software that controls a Twitter account via the Twitter API and can autonomously tweet and retweet) or by users who tweet the same thing repeatedly, we excluded tweets with the exact same content from the collection. To exclude ads and spam, tweets that contained URLs were also excluded from the collection. Further, a per-phrase within tweet exclusion process was employed. Non-Japanese phrases were excluded. Although hashtags (phrases written with a # symbol used to index keywords or topics on Twitter), emojis, and emoticons might provide us with useful information about Twitter users such as their intention and emotion, we excluded them to improve the accuracy of the target word extraction (using morphological analysis and dependent structure analysis described in the next subsection).

### 2.2. Preprocessing

#### 2.2.1. Target word extraction

We extracted the target words from each tweet text. The process of target word extraction depended on the NLP of Japanese sentences. The Japanese language is a language without word boundaries, and so a morphological analysis was performed using Juman++ (Tolmachev et al., 2018). This is the process equivalent to Lemmatization in English language processing. To find the target words that were referred from the verbs representing touch desire and touch avoidance, a dependent structure analysis was performed using the Kurohashi-Nagao parser (KNP) (Kawahara and Kurohashi, 2006). A case structure analysis was then performed on the identified words to determine the target word. Finally, to confirm that the target was a noun, we confirmed whether the word exists in the word2vec (Goldberg and Levy, 2014) model provided by chiVe (W2V, 2020). The numbers of tweets with extracted target words are shown in the fourth column in Table 1.

Since we noticed that some of the tweet text do not represent the desire or avoidance to physically touch targets. For example, the text “I want to touch mahjong tiles” does not necessarily mean the touch desire for the mahjong tile but the intention to play mahjong. These “noisy” targets should be excluded for analysis. We carefully excluded such target words from our dataset (see the details of exclusion in Supplementary Note 1). After all of these steps were completed, the target word was automatically translated into English using the Google Translate API. If the word was not appropriate for academic reporting, we reworded it into a more suitable one. The number of raw tweet texts and the total number of target words used for analysis are shown in the fifth column in Table 1.

#### 2.2.2. Classification of target words into animate and inanimate targets

Following our previous study (Ujitoko et al., 2021), we manually categorized the target words into six categories: “body part,” “animal,” “object,” “person,” “geometry,” and “temperature.” See the detailed reason for these categorization in the previous study (Ujitoko et al., 2021). Example words of the “body part” category are hand, hair, or buttock. Those of the “animal” category are cat or dog. Those of the “object” category are doorknob or money. Those of the “person” category are you or people. Those of the “geometry” category are line or ditch. Those of the “temperature” category are warmth or heat.

In this study, we defined the target words that belong to “body part,” “animal,” and “person” categories as animate targets and the target words that belong to “object” and “geometry” categories as inanimate targets. Since we find some target words belonging to the “temperature” category hard to classify, we looked at the tweet text to decide whether they were used in a way that was inanimate or animate.

In brief, we constructed four datasets of touch targets which were characterized by two touch queries and two animate/inanimate categories.

### 2.3. Quantity analysis

#### 2.3.1. Proportion of the tweet texts containing touch desire and touch avoidance

We investigated the effect of COVID-19 on changes in the number of tweet texts containing touch desire and touch avoidance. However, the number of tweet texts could be influenced by unintended factors such as changes in active user population or in the tweet-posting frequency of each user. To control these unintended factors, we calculated the proportion of the number of target words with respect to a rough estimate of the total number of tweets (hereinafter, we call the calculated proportion the “tweet proportion”). Since the monthly total number of tweets in Japan is not available publicly, we developed a way to estimate the total number of tweets (pseudo-baseline). We counted tweets containing the first Japanese symbol, “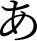 (a).” We randomly selected period of 30 s every in each 3 h period and used the sum of the tweets containing “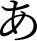 (a)” as a baseline number for each month. See the Supplementary Note 2 for the reason for adopting this counting method and the validation of this method. The number of pseudo-baseline tweets per month since August 2013 is shown in Supplementary Figure 1. We aggregated the pseudo-baseline and the tweet texts containing touch attitudes every third day to calculate three samples of tweet proportion for each month. For example, the first sample of tweet proportion of a month was composed of the data from the 1^*st*^, 4^*th*^, 7^*th*^ day, and so on.

#### 2.3.2. DID model on the quantity of the tweet texts

A challenge in estimating the effect of COVID-19 is to disentangle the effect of the pandemic from the long-term trend and its seasonality. Using animate targets as an example, the average tweet proportion of touch desire for animate targets declined by 23.8 % from 2013 to 2019 (Supplementary Figure 2A). In addition, before the pandemic (from 2013 to 2019), the average tweet proportion of touch avoidance for inanimate targets in February is 11.3 % smaller than in January (Supplementary Figure 2D). These data imply that a simple before-after comparison would not be able to detect the effect of COVID-19. That is, if the comparison is made between the tweet proportion before and during the COVID-19 outbreak, the result might capture the seasonal trend. Alternatively, if the comparison is made on the tweet proportion relative to past years in the same season, the result might be confounded by a long-term ascending or descending trend.

The common methods for the assessment of causal inference using panel data are a difference-in-differences (DID) analysis (see the review paper Wing et al., 2018 for an introduction) and an interrupted time-series analysis (Hartmann et al., 1980). While both analyzes are able to account for long-term trends, the interrupted time-series analysis cannot account for seasonal variation. In contrast, the DID can account for both seasonal and long-term trends.

**Figure 2:**
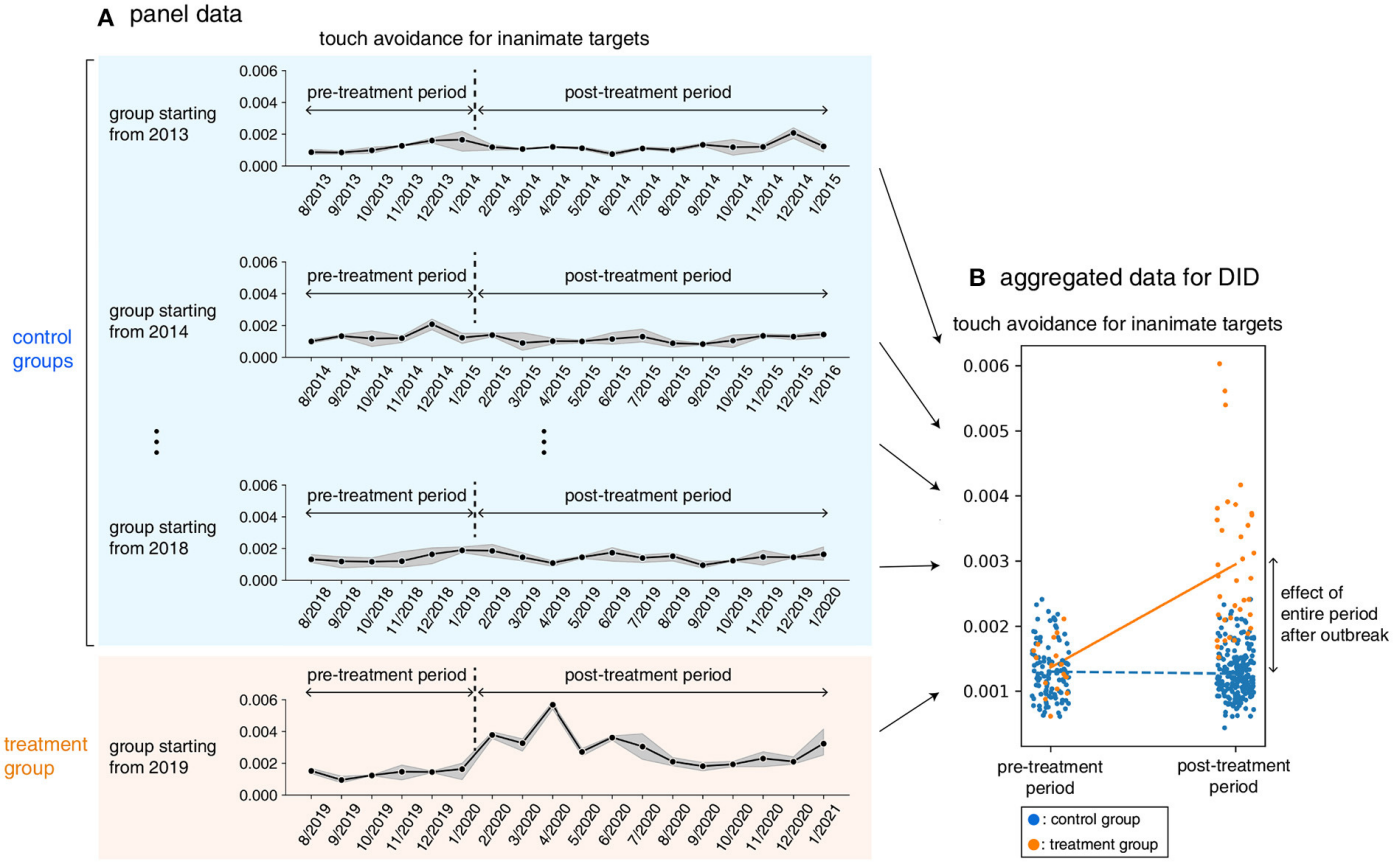
**(A)** Panel data comprised seven groups that started between 2013 and 2019. The group that started from 2019 is referred to as the “treatment group” (orange panel). The groups that started from 2013 to 2018 are referred to as the “control groups” (blue panel). The data of each group comprise 18 months (from August in the first year to January in the third year). Each group has a pre-treatment period (from August in the first year to January in the second year) and a post-treatment period (from February in the second year to January in the third year). **(B)** The panel data was aggregated for the pre-treatment and post-treatment periods. The difference between post-treatment and pre-treatment was calculated for both treatment and control groups. The difference between the treatment and control groups in the difference between the post- and pre-treatment periods was regarded as the effect of the entire period after the COVID-19 outbreak.

Here we explain how we applied the DID estimation method to our data. Figure 2A shows the panel data used in our analysis. We refer to the 18-month period from August 2019 to January 2021 (i.e., including the pandemic period) as the “treatment group,” and each period of 18 months starting from August 2013 up to that starting in August 2019 as a “control group.” Within each group, we refer to the period from August of the first year to January of the next year as the “pre-treatment period,” and the period from February in the second year to January in the third year as the “post-treatment period.” For example, in the group starting in 2013, the pre-treatment period denotes the period from August 2013 to January 2014 and the post-treatment period denotes the period from February 2014 to January 2015. The DID model compared the tweet proportion difference between the pre-treatment period and the post-treatment period in the treatment group with the difference between the corresponding pre-treatment and post-treatment periods in the control groups (see Figure 2B). By considering the relative difference in the corresponding pre-treatment and post-treatment periods, the model can cancel out the long-term trend and the seasonal effect.

In particular, we specified the following model:


(1) $$\begin{array}{l} Y_{gm}=\alpha Treat_{g}\times Post_{m}+\mu_{g}+\epsilon_{gm} \end{array}$$

where *Y*_*gm*_ denotes the tweet proportion in month *m* in group *g*. α is the parameter of interest, which denotes the impacts of the COVID-19 pandemic on the tweet proportion. *Post*_*m*_ is a binary variable that takes the value 1 if the month of observations is within the post-treatment periods. It takes a value of 0 if the month is within the pre-treatment period. *Treat*_*g*_ takes the value 1 if the group belongs to the treatment group and 0 if the group belongs to the control group. In this model, the tweet proportion difference between the pre-treatment and post-treatment periods in the control group serves as the control condition (counterfactual), after accounting for the difference of offsets across groups, with the assumption that we encounter only common shocks during the control and the treatment periods (i.e., during the pandemic). We include the group fixed effect, denoted by μ_*g*_. The group fixed effect is configured to control the underlying differences in the offsets among the previous six groups starting from 2013 up to 2018 (Wing et al., 2018). ϵ_*gm*_ denotes the error term.

The tweet proportion during the pandemic could vary across periods depending on the number of COVID-19 infections, or the people's responses and government health interventions in light of the outbreak. Specifically, Japan faced three large outbreak waves by February 2021 (Karako et al., 2021) (Supplementary Figure 3), and we might expect that the tweet proportion trend varies in each wave. Unlike the time-series interrupted analysis, the DID design enables us to estimate the time-varying effects flexibly. Therefore, we estimate the effect of the three waves with the following equation:


(2) $$\begin{array}{l} Y_{gm}=\;\alpha_{1st}(Treat_{g}\times First_{m})+\alpha_{2nd}(Treat_{g}\times Second_{m})\\ \;+\alpha_{3rd}(Treat_{g}\times Third_{m})+\mu_{g}+\epsilon_{gm} \end{array}$$

where *First*_*m*_, *Second*_*m*_, and *Third*_*m*_ denote dummy variables; *First*_*m*_ takes the value 1 during months corresponding to the first wave period (February to June in the second year), *Second*_*m*_ takes the value 1 during months corresponding to the second wave period (July to November in the second year) and *Third*_*m*_ takes the value 1 during months corresponding to the third wave period (December in the second year to January in the third year). In other periods these dummy variables take the value 0.

Our outcome variable of interest, tweet proportion, is a non-negative continuous value. Therefore, we use a Gamma distribution with the inverse link function. The adjusted model can be written as:


(3) $$\begin{array}{l} \frac{1}{Y_{gm}}=\;\alpha_{1st}(Treat_{g}\times First_{m})+\alpha_{2nd}(Treat_{g}\times Second_{m})\\ \;+\alpha_{3rd}(Treat_{g}\times Third_{m})+\mu_{g}+\epsilon_{gm} \end{array}$$

#### 2.3.3. Event-study approach

The assumption for the DID estimator to be valid is that the pre- and post-treatment periods in the treatment group and the same periods in the control groups would have parallel trends in tweet proportion in the absence of the pandemic. If this assumption were not satisfied, the estimated parameter would be biased because the results could be driven by systematic differences between the treatment and control groups rather than the event of interest. To assess whether the parallel trends assumption would be reasonable, we adopt the event-study approach by fitting the following equation (He et al., 2020; Leslie and Wilson, 2020; Tanaka and Okamoto, 2021):


(4) $$\begin{array}{l} \frac{1}{Y_{gm}}=\overset{18}{\underset{k=-6,k\neq -1}{\sum }}\alpha_{k}\left(Treat_{g}\times Month_{m,k}\right)+\mu_{g}+\epsilon_{gm} \end{array}$$

where *Month*_*m, k*_ is 1 if the month corresponds to *k*, where *k* = −1 is set to be a month in the pre-treatment period (January). If the month does not correspond to *k*, *Month*_*m, k*_ is 0. Intuitively, this causes a difference in tweet proportion between the treatment group and the control groups in each month relative to *k* = −1; we expect the treatment group and control group to have a similar tweet proportion before the disease outbreak becomes noticeable (*k* < 0) and we expect them to diverge after the outbreak (*k*≥0).

### 2.4. Quality analysis

#### 2.4.1. DID model on the quality of tweet texts

We also used DID to investigate the impact of the COVID-19 outbreak on the quality of tweet texts, that is, the probability distribution of target words in tweet texts (i.e., how the tweeted target words were changed before and after the COVID-19 pandemic). The method was the same as in the quantity analysis except that the variable of interest is not the tweet proportion, but the similarity of the probability distribution of the target words. The target word probability distribution of the pre-treatment period was used as a template, and the cosine similarity between the template and the probability distribution of each month of the same group was computed, and the similarity was used as a dependent variable. By using DID, we clarified whether tweet similarity increased or decreased after the outbreak (from February 2020 to January 2021) as compared to that of the pre-COVID-19 period (from August 2019 to January 2020). We controlled the long-term and seasonal effects by comparing the tweet similarities with those of the corresponding periods in previous years. A decrease in similarity indicates that the targets of each touch attitude changed after the COVID-19 outbreak.

Since the cosine similarity is also a non-negative continuous value, we use a Gamma distribution with the inverse link function. We used the same expressions (2), (3), and (4) to investigate the effect of the COVID-19 outbreak and the 1st–3rd waves, and to validate the common trend assumption.

#### 2.4.2. Analysis of what target differentiates the tweet quality

For the specific touch attitude at a specific period when there was a difference in quality in the above analysis by DID, we further analyzed what specific targets significantly increased or decreased in the probability distribution. We computed the difference of target words' probability distributions of the specific period, which is the period when there was a difference in quality in the above analysis by DID, between the treatment and control groups. The top 15 target words, which meant that people's touch attitude for them increased after the outbreak, and the bottom 15 target words, which meant that people's touch attitude for them decreased, were identified. We calculated 10,000 bootstrap samples of the differences and the one-tailed Bonferroni-corrected 95 % CI to test if the increase or decrease of the target words was significant. When the 95 % CI did not overlap zero, we concluded that the difference was not just due to chance.

In addition, we checked the raw tweet texts containing each target word that was different before and after the outbreak and considered the context wherein users tweeted the target word (see Supplementary Notes 3, 4). We referred to this check for the raw text as the “context check.”

## 3. Results

### 3.1. Changes in the quantity of tweet texts reflecting touch desire and touch avoidance

**Figure 3:**
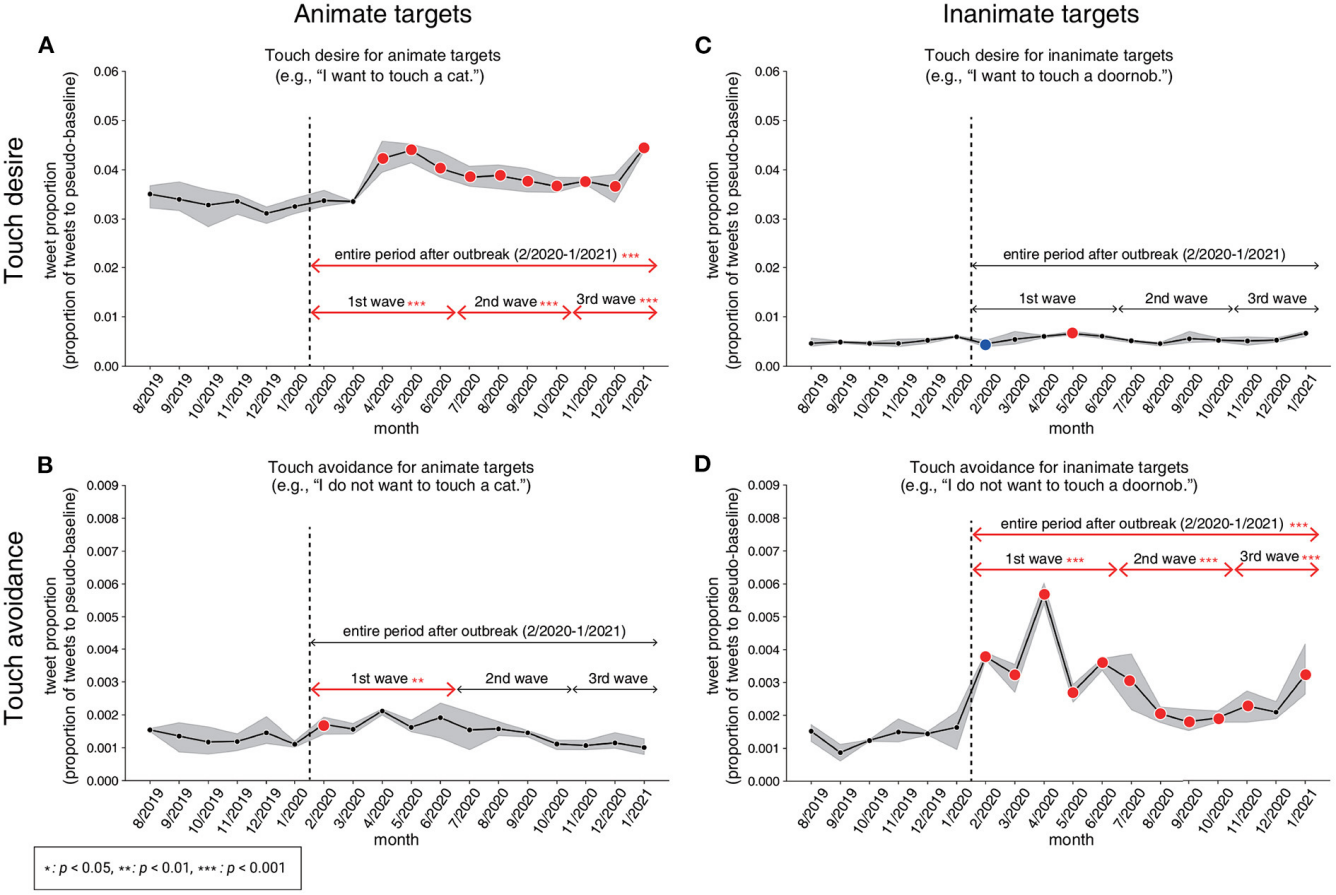
Changes in tweet proportion and the results of the DID and the event study. Results on touch desire **(A)** and touch avoidance **(B)** for animate targets. Results on touch desire **(C)** and touch avoidance **(D)** for inanimate targets. Lines represent the tweet proportion (the proportion of tweets relative to the pseudo-baseline) with shaded areas showing the 95% Confidence Interval (CI). The vertical dashed line indicates the timing of the outbreak. In addition, the effects of the entire period after the COVID-19 outbreak and the 1st–3rd waves are indicated (full results are presented in Supplementary Table 1). Red-colored arrows represent that the tweet proportion increased significantly during those periods. Blue-colored arrows represent that the tweet proportion decreased significantly during those periods. Also, red-colored and blue-colored points denote the results of the event study analysis. Red-colored points represent that the difference in tweet proportion between the focused month after the COVID-19 outbreak and the other 6 months just before the COVID-19 outbreak increased significantly relative to those in past years (2013–2018). Blue-colored points represent that the difference in tweet proportion between the focused month after the COVID-19 outbreak and the other 6 months just before the COVID-19 outbreak decreased significantly relative to those in past years (2013–2018). The decrease and increase in the tweet proportions were not significant in any month in the period before the COVID-19 outbreak, which supports the assumption for the parallel trends.

The changes in the proportion of tweets expressing touch desire and touch avoidance are shown in Figure 3. We confirmed that the assumption for parallel trends for DID is not violated during the period before the COVID-19 outbreak by showing that the decreases and increases in the tweet proportion were not significant for all months in the period before the COVID-19 outbreak (the event-study approach; see Section 2.3.3).

Our analyzes indicate that the proportion of tweet texts containing touch desire for animate targets (e.g., “hand,” “cat,” or “you”) has significantly increased in the entire period after the outbreak (Figure 3A), which was evidenced by a statistical test result in which the estimated coefficient was significantly larger than 0 (Supplementary Table 1 for more detail). In particular, the tweet proportion had a sudden increase in April 2020, which corresponds to the first SoE in Japan (State of Emergency; see also Supplementary Note 5). After that, although the number of COVID-19 infections repeatedly decreased and increased (i.e., the 2nd and the 3rd waves; see also Supplementary Figure 3), the tweet proportion remained constantly high. There is no statistically significant change in the proportions of tweets indicating touch avoidance for animate targets in the entire period after the outbreak (Figure 3B). However, we found a statistically significant change in the tweet proportions for touch avoidance in the 1st wave period (especially from February, right after the outbreak).

For inanimate targets (e.g., “doorknob,” “money,” or “water”), the tweet proportion containing touch desire did not change significantly in the entire period after the outbreak (Figure 3C). In contrast, tweet proportions containing touch avoidance significantly increased in the entire period after the outbreak (Figure 3D). Touch avoidance shows a clear increase during the 1st wave (particularly for April 2020), and then relatively decreased during the 2nd and 3rd waves as compared to the 1st wave. Indeed, we could not find a significant increase in December 2020 by the event study approach. This might indicate that the values are relatively similar to their previous values (i.e., old normal).

### 3.2. Changes in the quality of tweet texts reflecting touch desire and touch avoidance

**Figure 4:**
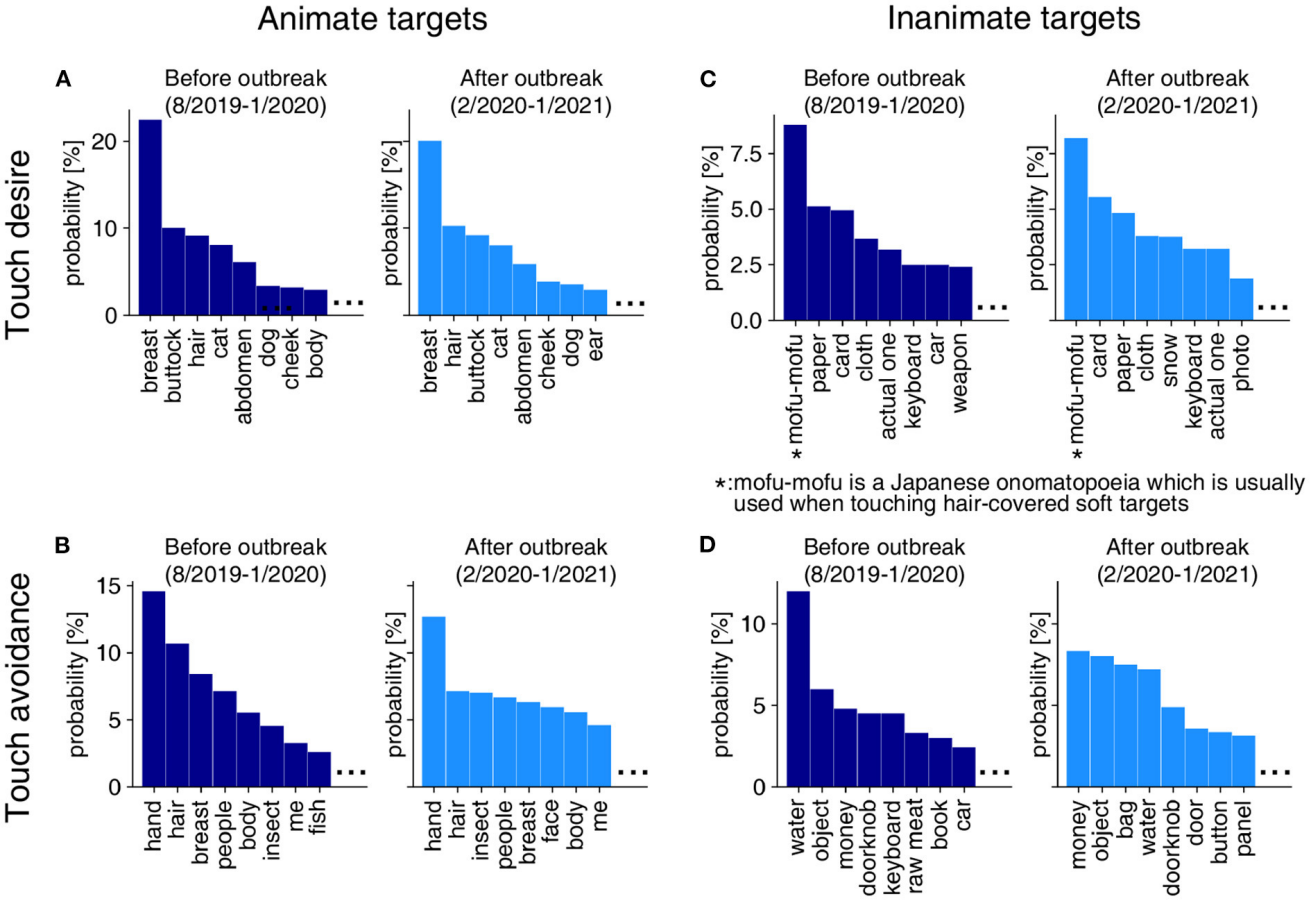
Probability distribution before and after the outbreak in terms of touch desire and touch avoidance for animate and inanimate targets. Results on touch desire **(A)** and touch avoidance **(B)** for animate targets. Results on touch desire **(C)** and touch avoidance **(D)** for inanimate targets.

Here we examined how the COVID-19 outbreak influenced the targets in tweet texts representing touch desire and touch avoidance (i.e., quality of tweet texts). Figure 4 shows the changes in the probability distribution of the targets of touch desire and touch avoidance. Taking animate targets for touch desire as an example, breast, buttock, and hair were the top target words both before and after the outbreak. However, the percentage of breasts and buttocks seemed to decrease after the outbreak.

**Figure 5:**
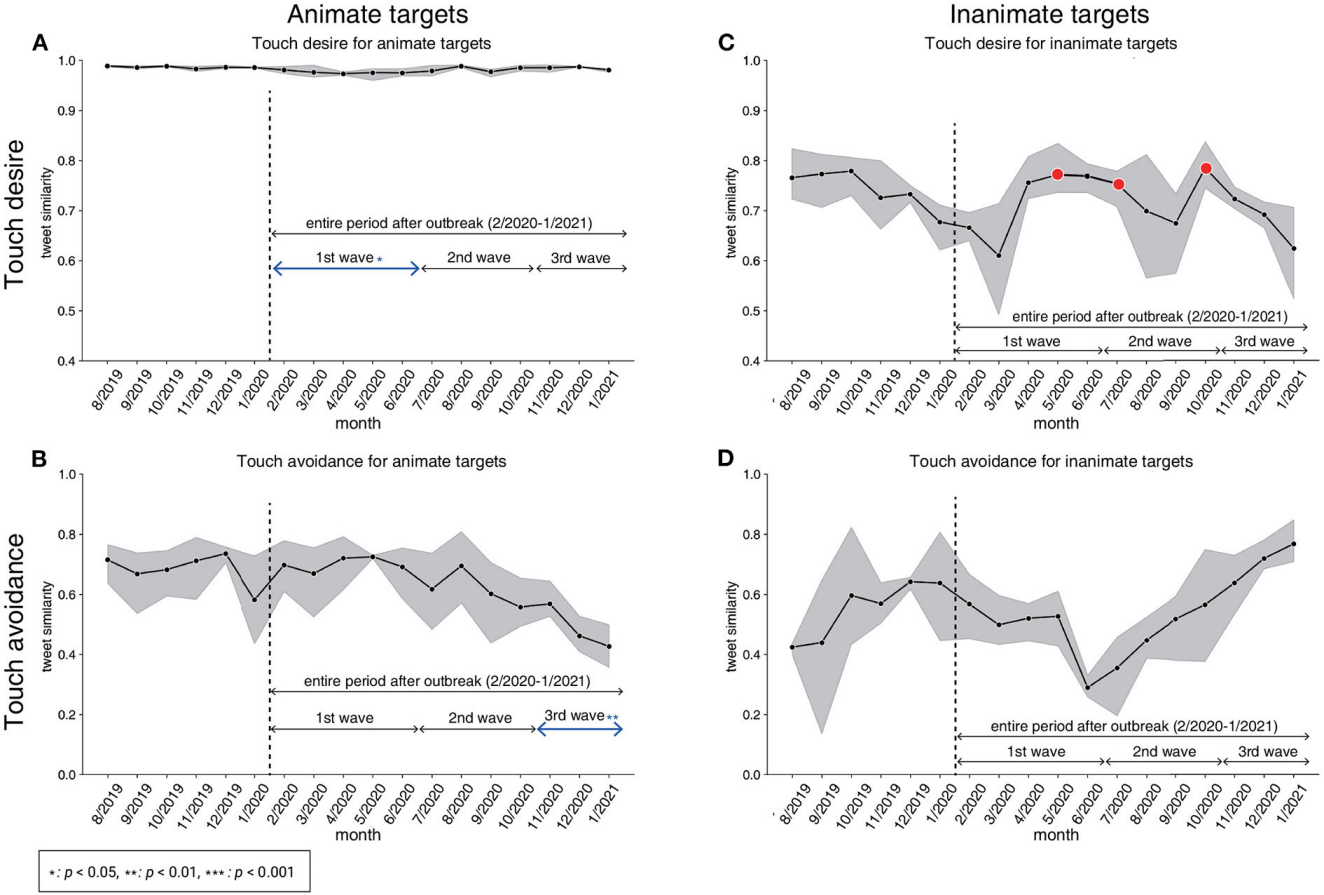
Variation of tweet similarity and the results of the DID and the event study. Results on touch desire **(A)** and touch avoidance **(B)** for animate targets. Results on touch desire **(C)** and touch avoidance **(D)** for inanimate targets. The difference of the tweet similarity before and after the outbreak was compared to the control period (i.e., corresponding periods in previous years). Lines represent the tweet similarity with shaded areas showing 95% CI. The vertical dashed line indicates the timing of the outbreak. In addition, the effect of the COVID-19 outbreak and the 1st–3rd waves are indicated. Full results are presented in Supplementary Table 2. Red-colored arrows represent that the similarity increased significantly during those periods. Blue-colored arrows represent that the similarity decreased significantly during those periods. Also, red-, and blue-colored points denote the results of the event study analysis. Red-colored points represent that the difference in the tweet similarity between the focused month after the COVID-19 outbreak and the other 6 months just before the COVID-19 outbreak increased significantly relative to those in past years (2013–2018). Blue-colored points represent that the difference in the tweet similarity between the focused month after the COVID-19 outbreak and the other 6 months just before the COVID-19 outbreak decreased significantly relative to those in past years (2013–2018). The increase and decrease in the tweet similarity were not significant in any month in the period before the COVID-19 outbreak, which supports the assumption for the parallel trends.

**Figure 6:**
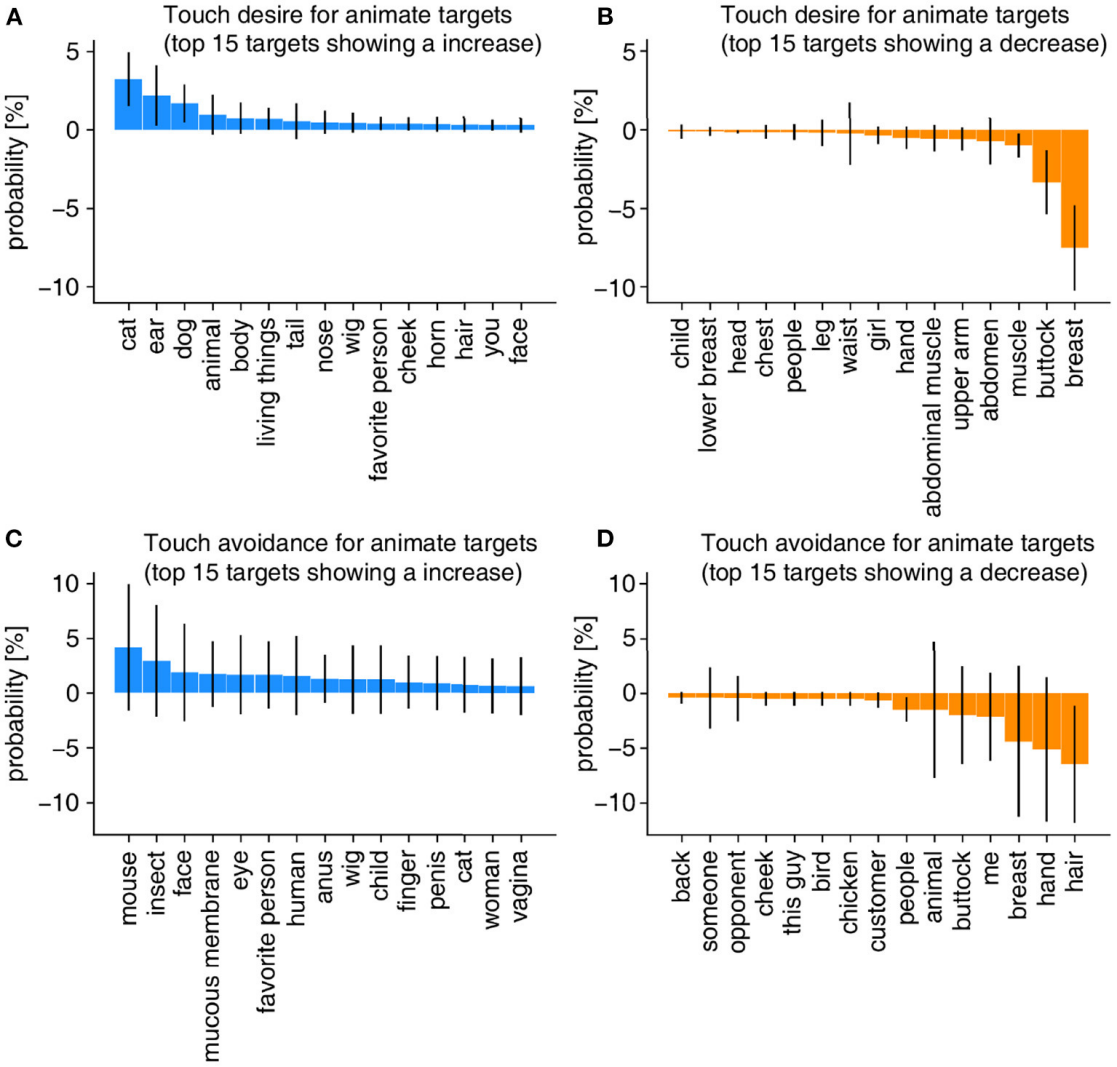
The difference in probability distributions for the top 15 and bottom 15 targets that exhibited a significant difference between the period before the outbreak and the period showing a significant change in the analysis of DID (Figure 5) are shown. **(A,B)** Touch desire for animate targets during 1st wave in 2020. **(C,D)** Touch avoidance for animate targets at 3rd wave period in 2020. The blue bars indicate the increased probability and the orange bars indicate the decreased probability. For each target, the calculated 95% CI with Bonferroni correction with bootstrap samples are shown in the figure.

To investigate if there is a relationship between the COVID-19 outbreak and the decreases or increases of the target words in the probability distribution, we examined the changes in the probability distributions before and after the outbreak. We calculated the cosine similarity between the probability distribution before the outbreak (i.e., the distribution in dark blue in Figure 4) and that for each month from August 2019 to January 2021. The changes in the similarity of the targets' probability densities for touch desire and touch avoidance are shown in Figure 5. As is the case in the quantitative analysis above, we used the DID estimation to clarify whether tweet similarity increased or decreased after the outbreak (from February 2020 to January 2021) as compared to that of the pre-COVID-19 period (from August 2019 to January 2020). We controlled the long-term and seasonal effects by comparing the tweet similarities with those of the corresponding periods in previous years. A decrease in similarity indicates that the targets of each touch attitude changed after the COVID-19 outbreak. The DID analysis revealed a significant change for the animate targets only (Figure 5). Figure 6 shows the difference in probability distributions for the top 15 and bottom 15 targets that showed a significantly large difference.

While the animate targets attracting touch desire did not change significantly in the entire period after the outbreak, there was a statistically significant change during the first wave (Figure 5A). The words “cat,” “ear,” and “dog” increased during 1st wave 2020 (Figure 6A). We reviewed the texts regarding “ear” and we found that at least 35.9% of the tweets suggested animals' ears (see Supplementary Note 3). Taken together with the increasing probability of the words “cat” and “dog,” these results suggest that the touch desire for animals increased in the 1st wave period. In contrast, the body parts such as breasts, buttocks, and abdomen where people can touch intimately (Suvilehto et al., 2015) became less frequently mentioned (Figure 6B).

While the occurrences of the animate targets eliciting touch avoidance did not change significantly in the entire period after the outbreak (Figure 5B), there was a significant change in the 3rd wave. The target “hair” decreased significantly in the 3rd wave period.

In order to check what situation users mentioned touch avoidance toward the hair, we checked the raw tweet texts. The context check of the texts including hair before the outbreak suggests that more than 60% of the occurrences of the target “hair” were used to indicate other people's hair in various situations such as a crowded train and a beauty salon (see Figure 6D and Supplementary Note 4).

## 4. Discussions

In this study, we extracted touch attitudes, i.e. touch desire and touch avoidance, from large-scale tweet texts of tweets using the method that we developed for Twitter analysis (Ujitoko et al., 2021). This analysis allowed us to identify the timings of the increase in touch desire for animate targets and in touch avoidance for inanimate targets. In this section, we will discuss the possible relationships between these results and “skin hunger” or awareness for infection control.

For animate targets (e.g., humans, body parts, etc.), our findings indicated a significant increase in the tweet proportion of touch desire after the outbreak, for the entire period and for all of the 1st–3rd waves (Figure 3A). This suggests sign of “skin hunger,” a public health crisis that has recently been featured in the media (Maham Hasan, 2021). The present study quantitatively observed “skin hunger” with time-series data for the first time. In particular, by incorporating the event study approach, we identified the increase starting from April 2020, which corresponded to the timing when SoE was first announced and social distancing was officially recommended in Japan. The tweet proportion of touch desire for animate targets remained high after April 2020, despite the fact that the number of daily COVID-19 infections had decreased. The results suggest that the Japanese people might not have enough physical contact with their family and friends due to the social distancing rules. Therefore, people kept on posting about their desire for touching animate targets on Twitter, regardless of the change in the number of daily COVID-19 infections.

We also found an increase in the touch desire for animals, especially dogs, and cats (see Figure 6). These results are consistent with the findings that pets could provide emotional support during the lockdown. It has been demonstrated that pets such as dogs or cats provided people with substantial support to mitigate the negative effects of lockdown in the UK and in Spain (Bowen et al., 2020; Ratschen et al., 2020). It is possible that people in Japan also tried to seek support from pets to alleviate the impact of stay-at-home requests.

Though the breasts or buttocks were the top targets of touch desire in the pre-COVID-19 period (see Figure 4A), their probabilities decreased during the COVID-19 pandemic. The reason for the decrease in breasts and buttocks as targets of touch desire is unclear but it might be related to a decrease in sex drive. Recent studies have shown that women in the UK and men in Italy have a tendency for low libido during the lockdown and quarantine, respectively (Cito et al., 2021; Wignall et al., 2021). In fact, the number of acts of sexual intercourse has decreased during quarantine in Italy (Cito et al., 2021). Interestingly, at the same time, there was a sharp increase in pornography searches in countries where COVID-19 is widespread (Zattoni et al., 2020). All the evidence points to the fact that the COVID-19 pandemic has changed people's sexual behavior.

As for the touch avoidance, our results show that there was a decrease in the touch avoidance for the animate target “hair” during the 3rd wave (from November 2020 to January 2021, Figure 6). From our context check (see Supplementary Note 4) on the tweet texts during the 3rd wave period compared with the previous years (2013 to 2019), we found that more than 60% of the “hair” mentioned in the context of touch avoidance is about avoiding touching other people's hair in various situations (e.g., in a crowded train or in a beauty salon). Our results might reflect the fact that these situations have decreased because of the stay-at-home requests.

For inanimate targets, our results showed a sudden increase in touch avoidance just after the outbreak and after that, it tended to return to the level of the pre-COVID-19 period (Figure 3D). The results indicate that people posted “I don't want to touch objects like door knobs and money” on Twitter less frequently after May 2020. It is unclear whether people stopped paying attention or whether people still paid attention but posted tweets less frequently. If the former assumption is correct, it is a warning sign for infection control. Since contact with contaminated surfaces is a possible means of COVID transmission (Van Doremalen et al., 2020), it is better for infection control to maintain high touch avoidance to contaminated surfaces.

While we found significant changes in the tweet proportions for inanimate targets eliciting touch avoidance after the outbreak, we didn't find any significant change in inanimate targets attracting touch desire. We speculated that the difference might be related to infection control measures; that is, while touch avoidance is directly related to infection control, touch desire has less direct association in the context of infection control. From the qualitative viewpoint, we didn't observe any change in quality for both the touch desire and avoidance for inanimate targets.

The touch attitudes extracted in the present study were consistent with some previous findings. For example, our data in Figure 4 showed touch avoidance for moist objects, such as water and raw meat, and objects that might spread the virus, such as doorknobs and money, which is in line with past findings that humans have the nature of avoiding damp things to prevent infection (Oum et al., 2011; Iwasa et al., 2020). In addition, an earlier finding that people have a preference for soft targets (Pasqualotto et al., 2020) was replicated in our study as we found that breast, buttocks, and mofu-mofu (one of the Japanese onomatopoeias which are usually used when touching hair-covered soft targets) are the top targets that people want to touch (see Figure 4).

One might assume that the targets of touch desire and touch avoidance are somewhat overlapping and the result of touch desire would negatively correlate with the result of touch avoidance. If this is the case, touch desire increases as touch avoidance decreases and vice versa. Inconsistent with the expectation, our data indicated that it is not the case (Figures 5, 3). For example, in Figure 3, the touch desire for animate targets increased from April 2020, but the touch avoidance for animate targets did not show the clear change at that timing. One possible explanation is that the overlap of animate targets between touch desire and touch avoidance was small in the present investigation. Therefore, the quantity or quality of targets of touch desire would not affect the quantity or quality of targets of touch avoidance, and vice versa. Indeed, our data showed that the targets of touch desire and the touch avoidance are different (Figure 4).

Human touch is often discussed in the framework of affective touch and discriminative touch (McGlone et al., 2014). We had considered classifying a touch as an affective one if the targets are a person or animals, and a touch as a discriminative one if the targets are objects. However, the relationship does not always hold true. For example, when one checks the skin temperature of another person by touch, the touch is not affective but discriminative. As another example, when we enjoy the softness of sweaters by touch, the touch is not discriminative but affective. As there are many possibilities between our touch intention and touch targets, it is difficult to accurately judge whether the touch mentioned in the tweet text is affective or discriminative. Therefore, we classified the targets into “animate” and “inanimate,” which provide an objective classification for the targets.

In our analysis, we grouped targets in the “body part” category (e.g., “head”), “person” category (e.g., “you”), and “animal” category (e.g., “cat”) into animate targets and we found a significant effect of the COVID-19 on touch desire toward animate targets. One might concern that people's touch desires for the “person” and “animal” might have independent temporal tendencies because they could be different in social and emotional aspects. Here, as a supplementary analysis, we investigated the correlation between the tweet proportions of touch desire in the person category and that in the animal category. The correlation is shown in Supplementary Figure 4. The Spearman's rank correlation coefficient was 0.68 (*p* < 0.001). The correlation indicates that the touch desires for “person” and “animal” have similar tendencies and it is reasonable to group them together.

## 5. Conclusion, contributions, and limitations

Using large-scale Japanese Twitter posts, we found that people's desire for touching the human body and pet animals increased significantly after the COVID-19 outbreak and remained high afterward. In contrast, the avoidance of touching everyday objects (e.g., doorknobs and money) increased immediately after the outbreak but gradually returned to the pre-COVID-19 levels. Our findings manifest the impact of COVID-19 on human touch behavior. Most importantly, they highlight the sign of “skin hunger,” a public health crisis due to social distancing, and call attention to the trend that people are becoming less aware of infection control as COVID-19 persists.

The method developed in this study should contribute to the analysis not only for the impact of the COVID-19 pandemic, but also for the changes in people's attitudes corresponding to major incidents such as artificial or natural disasters for policy making. The continuous tracking of touch attitudes using our method will also provide valuable insights into various disciplines of haptic research such as haptic engineering, haptic science, and haptic marketing. For example, the ongoing “skin hunger” would make people recognize the importance on the “touch” in the virtual world, and thus social touch technology (Huisman, 2017) which provides mediated social touch with haptic feedback will play an important role for the demand. The decrease in touch avoidance for infection control even while the COVID-19 pandemic continues suggests the necessity of mid-air haptic technology (Rakkolainen et al., 2020) that prevents users from touching contaminated surfaces regardless of users' touch attitude toward such surfaces. Also, when investigating human haptic functions and abilities in haptic science research, changes in touch attitudes over time may also need to be taken into account. This is because human's haptic abilities and functions may be correlated with changes in touch attitudes, as suggested by the fact that touch avoidance of pathogens may have formed part of humans' haptic preference for objects (Oum et al., 2011; Iwasa et al., 2020). Finally, in the field of haptic marketing, it is known that people use touch information on products in stores to make purchase decisions (Peck and Childers, 2003). However, if touch avoidance increases again, for example, due to the spread of a COVID-19 variant, people will be less likely to benefit from this touch information. Therefore, it would be important to consider presenting haptic features known to influence consumer behavior in another way (e.g., verbal or visual information) to support consumer decision-making.

We acknowledge the limitations of this study. The results were obtained by analyzing the posted Japanese texts on Twitter, so there may be biases in generalizing our findings. First, we focused on texts tweeted in Japanese, and thus the results obtained in this study are assumed to be Japanese-specific. Indeed, the roles of touch depend on the culture (Duarte and e Silva, 2020). For example, in Italy, a hug and kiss on each cheek are considered a common and acceptable form of greeting. In contrast, in Japan, a proper greeting comprises a bow and the absence of any physical contact (McDaniel and Andersen, 1998; Finnegan, 2002). Thus, the results would be different even when the same method is applied to different cultures because of the difference in public health, economic, and social contexts. Comparison studies of the COVID-19 impacts on people's touch desire and avoidance between Japanese and people in other countries would allow us to deepen our understanding of the effect of cultures on our results.

In addition, this study analyzed only self-reports by Twitter users but not by those who do not use Twitter. For example, the distribution of region, age, and gender in Twitter seems to be skewed relative to the true distribution of Japanese people (see the age and gender distribution in Japan in December 2018 in Supplementary Table 3). The spread of infection differs depending on the region, age, and gender, and it might affect the tweet texts, so it should be noted that the results are skewed. Further, although we assumed that the Twitter user population was unchanged for all periods in our data, the assumption cannot be warranted.

Moreover, this study focuses on tweet texts and it means that touch desire and touch avoidance in this study was only the specific part of touch desire and touch avoidance that Twitter users have. Those touch desire and touch avoidance were what Twitter users wanted to share with the public. Further, the difference in the ease of posting tweet texts might bias our results in this study. We characterized touch desire and touch avoidance by analyzing the target words based on specific queries. It is possible that the results might be affected by how casually the target word can be tweeted. Also, the selection of queries for extracting tweet texts could affect the result. We tried other query candidates by using other verbs (e.g. “like to touch” or “feel good to touch”) than “want to touch,” but it turned out that those queries only resulted in a small number of tweets. Therefore, we decided the query “want to touch” and “don't want to touch,” which extracted a large number of tweets, would be the best for a time-series analysis about touch desire and touch avoidance.

Even though our data might be biased due to the aforementioned factors, we believe that our results are valuable for getting insight into how COVID-19 affected our touch attitudes. Our study is the first report that Twitter texts allow us to track the changes in people's touch attitudes due to COVID-19. Our findings can contribute to understanding human touch behavior, improving mental health care, and policy-making. For the future work, it is important to understand the user's intentions and the emotional states that underlie these touch attitudes. Although the current analysis methods can't estimate the intentions and emotional states from Japanese text data reliably, it is expected that the analysis will be possible in the future to help us understand people's touch attitudes toward the targets they want to touch (or not want to touch).

## Data availability statement

The original contributions presented in the study are included in the article/Supplementary material, further inquiries can be directed to the corresponding author.

## Author contributions

YU, TY, H-NH, and YB conceptualized the study. YU, TY, and H-NH carried out planning and prepared the first draft of the report, which was revised by all authors. YB retrieved the dataset. YU preprocessed the dataset and carried out the statistical analysis. All authors contributed to the article and approved the submitted version.

## Conflict of interest

Authors YU, TY, and H-NH were employed by Nippon Telegraph and Telephone Corporation. The remaining author declares that the research was conducted in the absence of any commercial or financial relationships that could be construed as a potential conflict of interest.

## Publisher's note

All claims expressed in this article are solely those of the authors and do not necessarily represent those of their affiliated organizations, or those of the publisher, the editors and the reviewers. Any product that may be evaluated in this article, or claim that may be made by its manufacturer, is not guaranteed or endorsed by the publisher.
