# Supplementary material for: Tracking changes in touch desire and touch avoidance before and after the COVID-19 outbreak
Source: Front Psychol. 2022 Dec 1;13:1016909. doi: 10.3389/fpsyg.2022.1016909 (PMC9751362; doi:10.3389/fpsyg.2022.1016909)
Supplement: Supplementary file 1 [file Data_Sheet_1.pdf]

Supplementary Information for

"Tracking changes in touch desire and touch avoidance before and after the COVID-19 outbreak"

Yusuke Ujitoko[1], Takumi Yokosaka[1], Yuki Ban[2], Hsin-Ni Ho[1]
[1]NTT Communication Science Laboratories, [2] The University of Tokyo

Correspondence: Yusuke Ujitoko yusuke.ujitoko@gmail.com

Contents:

Supplementary Notes 1-5
Supplementary Figures 1-4
Supplementary Tables 1-3

Supplementary Notes

Supplementary Note 1: Context check of the tweet texts and exclusion of them if they do not represent touch attitude.

We conducted a context check of the tweet texts containing target words, and we noticed that there were “noisy” texts that were not related to touch attitude. To exclude such noisy texts, it would be best if automated exclusion to check texts were possible, but it was difficult to implement a system that could accurately judge texts as being noise or not automatically. In contrast, manual inspection of all of the tweets would be accurate but too time-consuming to apply. Thus, we adopted a third option; we configured a noisy target word list for each query. In that noisy target word list, the target words that would be excluded were listed. Next, we excluded the tweets that contained such noisy target words included in the list. Note that if a tweet containing a target word could not always be determined to be noisy, the target word was not added to the noisy target word list. In other words, the target word in some cases could not be excluded as noise. After defining the noisy target word list, we excluded tweets with target words that were included in the list.

Supplementary Note 2: Construction of the pseudo-baseline.

In order to know the rough estimate of relative differences in total tweets day by day, we randomly selected period of 30 seconds every in each three hour period on all days and counted the tweets containing the first Japanese symbol “あ(a)” as a pseudo-baseline number for each day. The pseudo-baseline needs to reflect the month-wise number of tweet texts as accurately as possible. To realize this, the definition of query and also the definition of posting timing of the tweet texts that are collected is important.

If the query has some meaning such as “雪(snow)”, the number of tweets acquired relative to the total number of tweets may fluctuate due to some event. For example, if the query is configured to be “雪(snow)”, the number of tweets may fluctuate due to seasonality and it does not reflect the total number of tweets. Therefore, we required a query that should be separated from the meaning, and thus we chose to count "あ(a)" which is just the first symbol of all 50 Japanese kana symbols.

Since the number of days fluctuates from month to month, we counted on all days of the month so that collected tweet texts reflect the number of tweets in each month as accurately as possible. We collected texts every three hours to alleviate the effect of the hourly variation of the frequency of posted tweet texts. We thought that randomization was necessary for the minimum unit of timing, and thus we selected a period of 30 seconds every three hour period.

The method of using pseudo-baseline as a rough estimation of the total number of tweets is developed in this study and thus we would like to validate this method. We would like to validate the pseudo-baseline in terms of yearly or monthly total tweets in Japan, but there is no ground truth for that value. (If there is, the metric of pseudo-baseline is unnecessary.) Here, we

estimate the reliability indirectly by evaluating hourly aggregated pseudo-baseline since we found the figure of the ground truth in a site (https://www.vicinitas.io/blog/twitter-social-media-strategy-2018-research-100-million-tweets). They collected tweet texts in Japan for 33 days (from 2nd January, 2018 to third February, 2018). We calculated the Pearson's correlation between their hourly change data and our pseudo-baseline's hourly change data, and the correlation coefficient was 0.97 (p<0.001). This suggests the validity of pseudo-baseline.

Supplementary Note 3: Context check of the tweet texts in the case of touch desire on animate targets.

We found that "ear" was the second top-ranked word in the difference of probability distribution in the 1st wave period before and after the outbreak. We listed all the tweet texts during 1st wave in 2020 in which the "ear" was the target of touch desire to check the contexts behind such tweet texts. We noticed that tweets frequently mentioned ear belonging to animals such as dogs or cats. We calculated the probability that the ear in the tweet texts implies the animal's ear. The probability of tweet texts implying an animal's ear was 37.5% of all 64 tweet texts while that of tweet texts implying a human ear was 14.0%. Note that some tweets do not reveal the owner of the ear which was the target of touch desire, and thus it seems that more than 37.5% of tweet texts actually indicated the touch desire toward animal ears.

Supplementary Note 4: Context check of the tweet texts in the case of touch avoidance on animate targets.

We found that the probability of "hair" was significantly decreased in the 3rd wave period after the outbreak. We listed all the tweet texts in which "hair" was the target of touch avoidance before the outbreak and speculated on the reason why tweets on hair touch avoidance were usually posted before the outbreak. We found that the hair means human hair and most of the tweet texts indicate that the hair belongs to other people. We examined the owners of the hair in the tweet texts and found that in more than 60% of the cases the hair belongs to other people. We found the situations where touch avoidance of other people's hair occurred vary (e.g., in a crowded train or in a beauty salon). We speculate that these situations might have decreased after the outbreak because people hesitated to go out.

Supplementary Note 5: State of Emergency Declaration in Japan.

On 7 April 2020, the government proclaimed a one-month state of emergency for Tokyo and the prefectures of Kanagawa, Saitama, Chiba, Osaka, Hyogo, and Fukuoka. On 16 April 2020, the declaration was extended to the rest of the country for an indefinite period. By the end of May, in most prefectures, the daily new COVID-19 infections were brought down to below ten. In line with this, the state of emergency was lifted from prefectures where the infection situation was

relatively mild on May 14, 2020, and nationwide on May 25, 2020. The 1st wave outbreak period (see Supplementary Figure 3) includes the period of the first state of emergency.

During the 2nd wave outbreak period, there was no declaration of a state of emergency.

At the outbreak of the 3rd wave, a second state of emergency was declared. On 7 January 2021, the government declared a state of emergency for Tokyo and the prefectures of Chiba, Saitama, and Kanagawa, effective from 8 January until 7 February. On 13 January, the region to which the state of emergency applied was expanded to 11 cities including Tochigi, Gifu, Aichi, Kyoto, Osaka, Hyogo, and Fukuoka. On February 2, the Japanese government decided to extend the COVID-19 state of emergency in 10 of the 11 prefectures where it was ongoing (excluding Tochigi) for one month until March 7. On March 5, it was announced that the state of emergency would be extended until 21 March for Saitama, Chiba, Tokyo, and Kanagawa. On 21 March, the state of emergency was lifted.

Supplementary Figures

Supplementary Figure 1: Number of pseudo-baseline tweets per month.

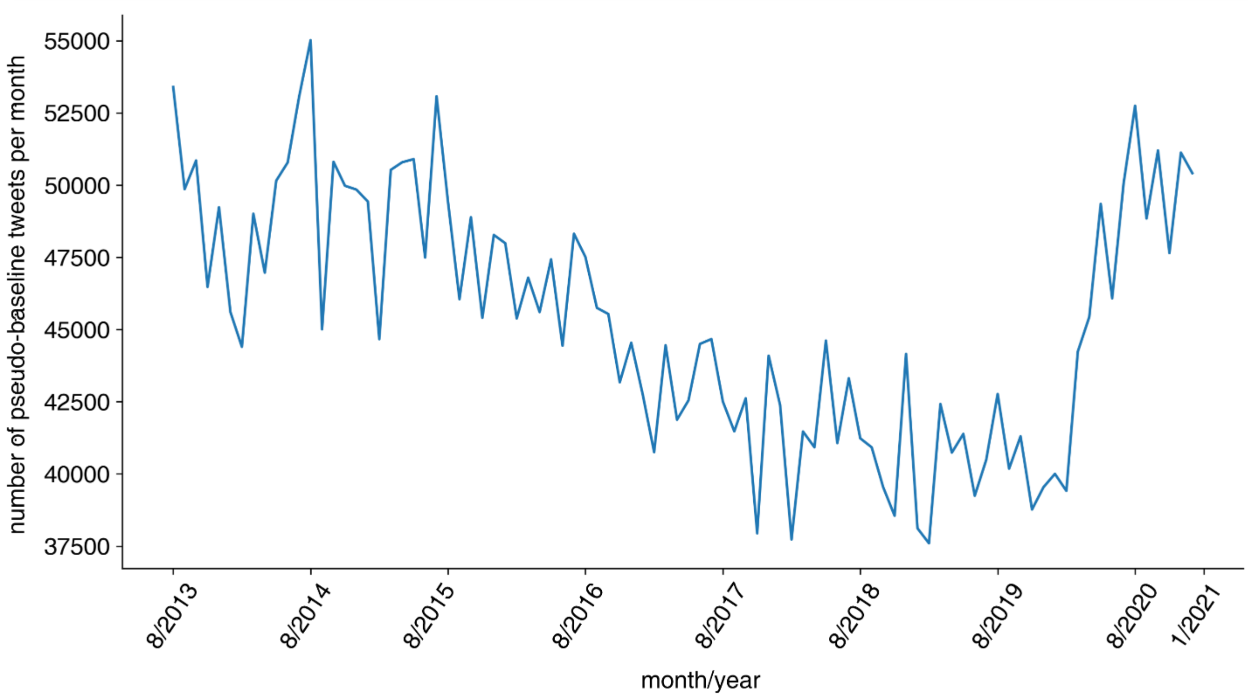

The blue line represents the number of pseudo-baseline tweets per month since August 2013. The number of pseudo-baseline tweets indicates the reference information for how many tweet texts are posted. Since Twitter doesn't publish the number of tweets in Japan, we defined our own baseline value. We counted tweets containing the first Japanese symbol, "a". We randomly selected a period of 30 seconds every three hour period on all days and used the sum of the tweets containing “a” as a pseudo-baseline number for each day. The number of pseudo-baseline tweets increased sharply after the COVID-19 outbreak. This is consistent with the report that indicates a significant increase in internet use after the outbreak (Nimrod 2020).

Nimrod, G. Changes in Internet Use When Coping With Stress: Older Adults During the COVID-19 Pandemic. Am. J. Geriatr. Psychiatry 28, 1020–1024 (2020).

Supplementary Figure 2: Long-term trends and seasonality of animate/inanimate target words.

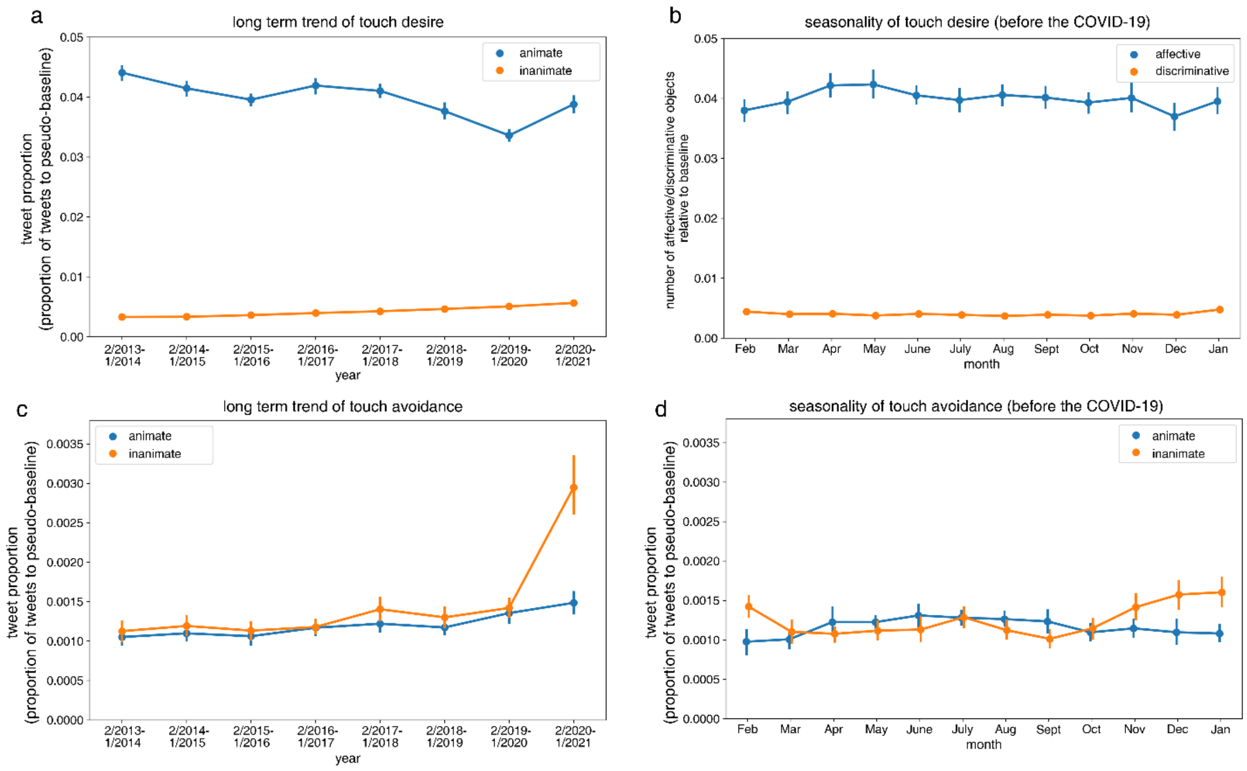

a, b, Long term trend **(a)** and seasonality **(b)** for touch desire for animate/inanimate targets. **c, d,** Long term trend **(c)** and seasonality **(d)** for touch avoidance for animate/inanimate targets. The values for each month in the seasonality graph **(b)(d)** were calculated using data from 2/2013 to 1/2020. The error bars denote 95% CI.

Supplementary Figure 3: Number of daily COVID-19 infections in Tokyo.

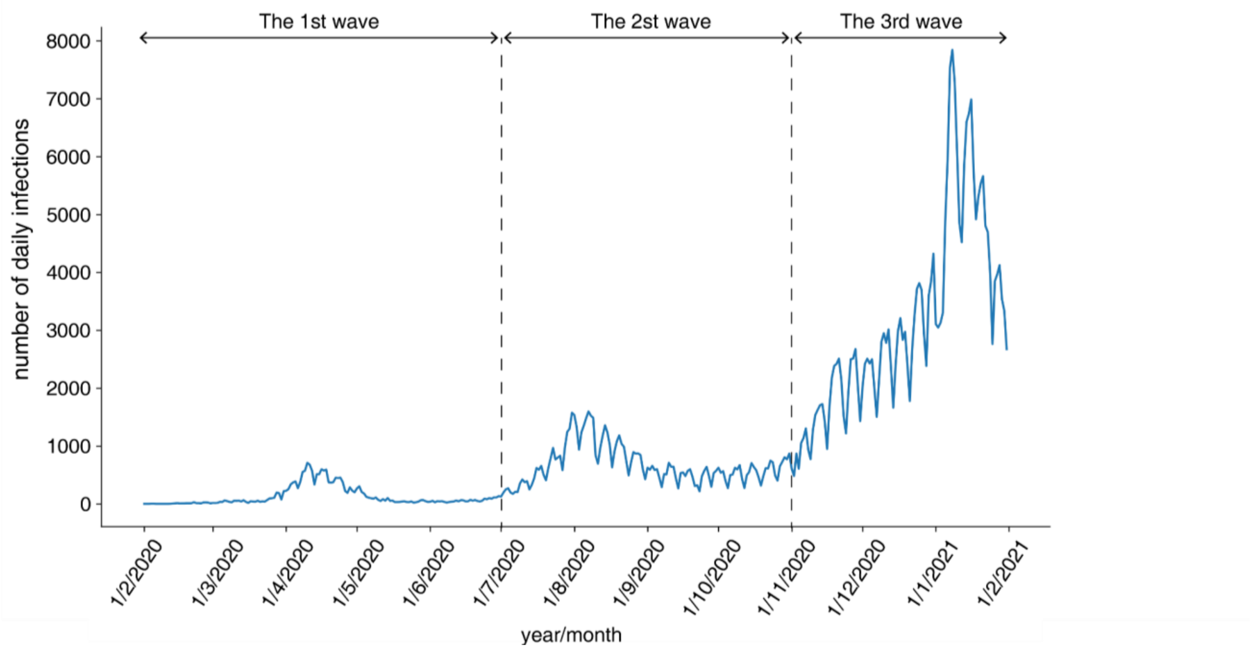

The blue line represents a number of COVID-19 infections. The source of information is Ministry of Health, Labour and Welfare Open Data.

Open Data. *Ministry of Health, Labour and Welfare* https://www.mhlw.go.jp/stf/covid-19/open-data.html.

Supplementary Figure 4: Correlation of tweet proportions between animal and person categories.

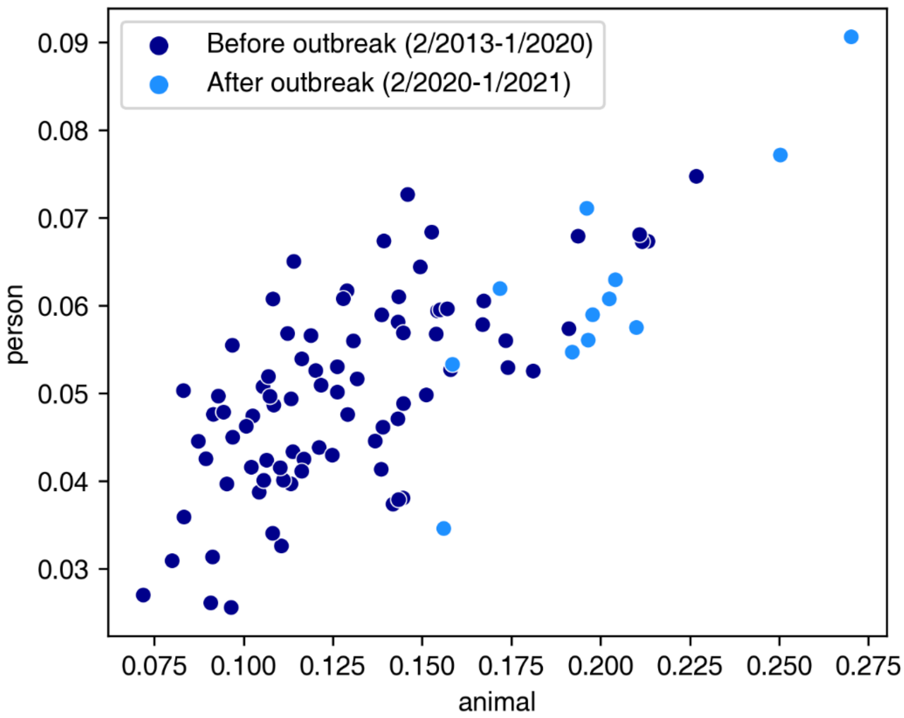

The dark blue plot represents the tweet proportion before outbreak and the light blue one represents that after outbreak. The x-axis shows the tweet proportion of touch desire for animal category. The y-axis shows the tweet proportion of touch desire for person category. The spearman's rank correlation coefficient was 0.68 ($p < 0.001$).

Supplementary Tables

Supplementary Table 1: Full results of the effects of COVID-19 pandemic on tweet proportion using DID.

	Touch desire		Touch avoidance	
	Animate targets	Inanimate targets	Animate targets	Inanimate targets
Treat * Entire period after outbreak	-5.13<br>(-6.67~-3.59)	1.77<br>(-19.79~23.32)	-52<br>(-175~71)	-411<br>(-528~-294)
Treat * First wave	-4.84<br>(-6.64~-3.04)	-4.74<br>(-29.6~20.1)	-177<br>(-309~-44)	-536<br>(-651~-421)
Treat * Second wave	-4.45<br>(-6.37~-2.52)	4.24<br>(-23.5~32.0)	18<br>(-135~171)	-361<br>(-492~-229)
Treat * Third wave	-6.52<br>(-8.58~-4.47)	9.7<br>(-18.7~38.1)	182<br>(-16~380)	-226<br>(-356~-97)
Group Fixed Effect	Yes	Yes	Yes	Yes
Objs	378	378	378	378

Each column shows the appropriate regression, giving the estimated coefficient and the 95% confidence interval in parentheses. The results correspond to Fig.1. Red-colored font indicates that the effect of the COVID-19 pandemic on the period was significant.

Supplementary Table 2: Full results of the effects of COVID-19 pandemic on tweet similarity using DID.

	Touch desire		Touch avoidance	
	Animate targets	Inanimate targets	Animate targets	Inanimate targets
Treat * Entire period after outbreak	0.002<br>(-0.004~0.008)	-0.03<br>(-0.13~0.08)	0.03<br>(-0.14~0.20)	-0.29<br>(-0.68~0.11)
Treat * First wave	0.008<br>(0.001~0.015)	-0.02<br>(-0.14~0.11)	-0.190<br>(-0.384~0.003)	-0.19<br>(-0.68~0.29)
Treat * Second wave	0.001<br>(-0.006~0.009)	-0.09<br>(-0.22~0.04)	0.06<br>(-0.16~0.28)	-0.51<br>(-1.05~0.02)
Treat * Third wave	-0.007<br>(-0.015~0.001)	0.05<br>(-0.10~0.20)	0.46<br>(0.18~0.74)	-0.23<br>(-0.67~0.22)
Group Fixed Effect	Yes	Yes	Yes	Yes
Objs	378	378	378	378

Each column shows the appropriate regression, giving the estimated coefficient and the 95% confidence interval in parentheses. The results correspond to Fig.2. Red-colored font indicates that the effect of the COVID-19 pandemic on the period was significant.

Supplementary Table 3: Age and gender distributions of Twitter users in Japan in December 2018.

	Male	Female
15-19	6.9%	9.7%
20-29	8.3%	13.8%
30-39	7.5%	12.7%
40-49	10.2%	8.0%
50-59	5.8%	8.8%
60-	5.0%	3.3%

The source is the site HumbleBunny (https://www.humblebunny.com/ja/japans-top-social-media-networks-for-2019/) and more up-to-date information is not available.
